# Topsoil and subsoil bacterial community assemblies across different drainage conditions in a mountain environment

**DOI:** 10.1186/s40659-023-00445-2

**Published:** 2023-06-24

**Authors:** Constanza Aguado-Norese, Valentina Cárdenas, Alexis Gaete, Dinka Mandakovic, Javiera Vasquez-Dean, Christian Hodar, Marco Pfeiffer, Mauricio Gonzalez

**Affiliations:** 1 Millennium Institute Center for Genome Regulation, Santiago, 7830490 Chile; 2 Bioinformatic and Gene Expression Laboratory, INTA-Universidad de Chile, Macul, Chile; 3 Departamento de Ingeniería y Suelos, Facultad de Ciencias Agronómicas, Universidad de Chile, Santiago, Chile; 4 GEMA Center for Genomics, Ecology and Environment, Universidad Mayor, Santiago, Chile; 5 Bioinformatic and Biostatistic Laboratory for Functional Genomics, INTA-Universidad de Chile, Macul, Chile

**Keywords:** Floodplain, Central Andes, High Mountain microbiome, Soil microbial community, Mediterranean climate, Pedology, Alpine soils

## Abstract

**Background:**

High mountainous environments are of particular interest as they play an essential role for life and human societies, while being environments which are highly vulnerable to climate change and land use intensification. Despite this, our knowledge of high mountain soils in South America and their microbial community structure is strikingly scarce, which is of more concern considering the large population that depends on the ecosystem services provided by these areas. Conversely, the Central Andes, located in the Mediterranean region of Chile, has long been studied for its singular flora, whose diversity and endemism has been attributed to the particular geological history and pronounced environmental gradients in short distances. Here, we explore soil properties and microbial community structure depending on drainage class in a well-preserved Andean valley on the lower alpine vegetation belt (~2500 m a.s.l.) at 33.5˚S. This presents an opportunity to determine changes in the overall bacterial community structure across different types of soils and their distinct layers in a soil depth profile of a highly heterogeneous environment.

**Methods:**

Five sites closely located (<1.5 km) and distributed in a well preserved Andean valley on the lower alpine vegetation belt (~2500 m a.s.l.) at 33.5˚S were selected based on a pedological approach taking into account soil types, drainage classes and horizons. We analyzed 113 soil samples using high-throughput sequencing of the 16S rRNA gene to describe bacterial abundance, taxonomic composition, and co-occurrence networks.

**Results:**

Almost 18,427 Amplicon Sequence Variant (ASVs) affiliated to 55 phyla were detected. The bacterial community structure within the same horizons were very similar validating the pedological sampling approach. Bray-Curtis dissimilarity analysis revealed that the structure of bacterial communities in superficial horizons (topsoil) differed from those found in deep horizons (subsoil) in a site-specific manner. However, an overall closer relationship was observed between topsoil as opposed to between subsoil microbial communities. Alpha diversity of soil bacterial communities was higher in topsoil, which also showed more bacterial members interacting and with higher average connectivity compared to subsoils. Finally, abundances of specific taxa could be considered as biological markers in the transition from topsoil to subsoil horizons, like Fibrobacterota, Proteobacteria, Bacteroidota for shallower soils and Chloroflexi, Latescibacterota and Nitrospirota for deeper soils.

**Conclusions:**

The results indicate the importance of the soil drainage conditions for the bacterial community composition, suggesting that information of both structure and their possible ecological relationships, might be useful in clarifying the location of the edge of the topsoil-subsoil transition in mountainous environments.

**Supplementary Information:**

The online version contains supplementary material available at 10.1186/s40659-023-00445-2.

## Background

Mountain soils cover 22-27% of the Earth’s total soil surface [1]. These soils are distributed across a wide range of latitudes, longitudes, and altitudes and comprise various habitats like topsoil, subsoil, biological soil crust, permafrost, and snow. The wide range of altitudes and slope aspects allows for an extraordinary diversity of physico-chemical features compared to nearby lowlands [2]. Since soil microbial community diversity and structure are largely driven by local environmental features and biotic factors [3–5], the distinctive physico-chemical diversity within mountain systems is expected to display a significant microbial diversity. Thus, soil microorganisms in mountainous environments could be considered as repositories of biodiversity and providers of ecosystem services [6, 7], including microorganisms that might be exploited for biotechnological purposes [8].

As the altitude, depth exert influences on microbial communities by shaping various habitat features. Factors such as pH, temperature, water availability, oxygen levels, and nutrient availability are known to vary with depth, thereby impacting the composition of microbial populations. For instance, soil microbial diversity has been described as decreasing with depth [9, 10]. While the relationship of soil depth on microbial communities has been widely studied [8, 11], a soil horizon based on a pedological feature sampling approach has rarely been used in environmental microbiology [12–14].

**Fig. 1:**
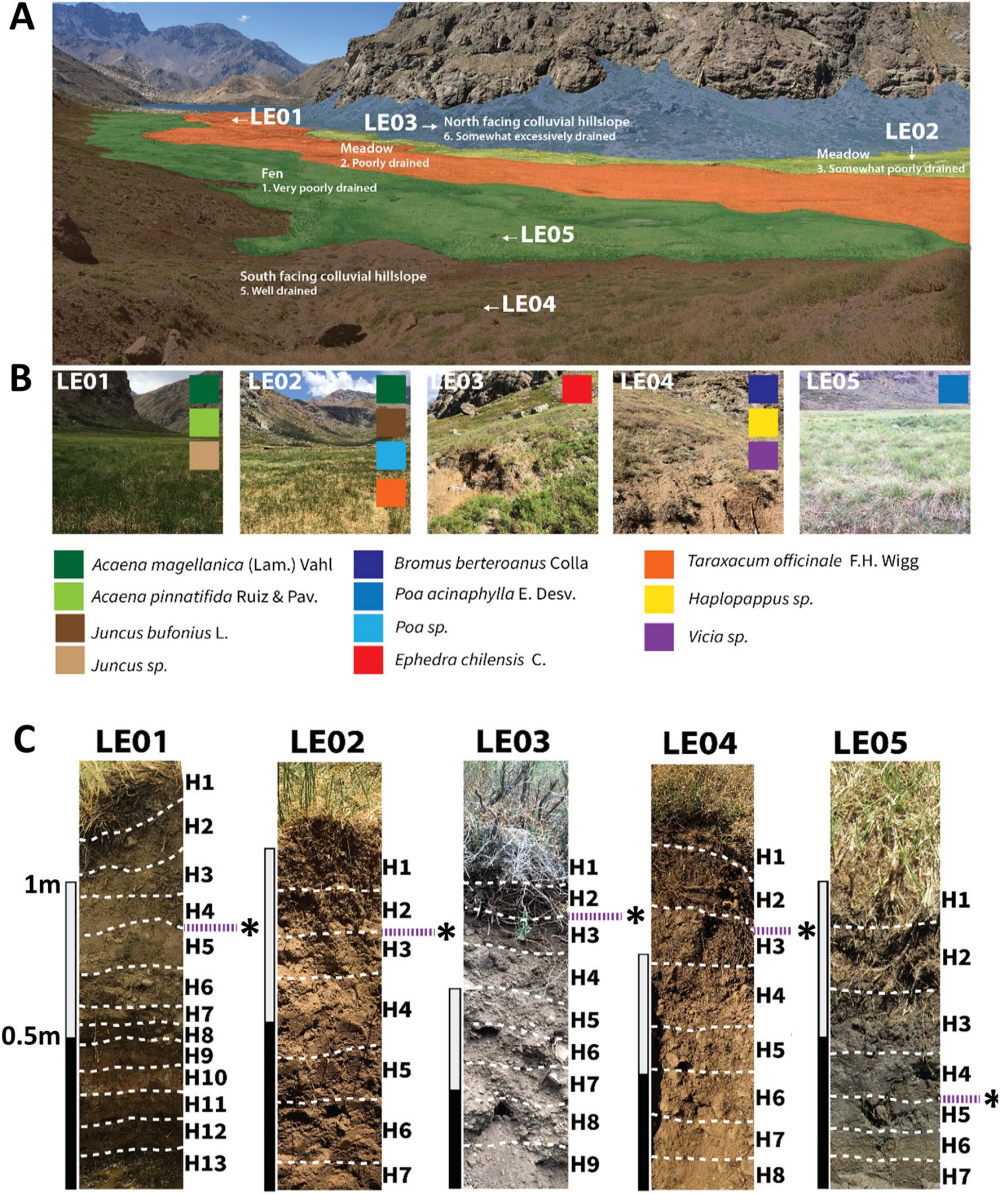
Location of the study sites and sampling strategy.** A**. Geomorporhic distribution of the soil covered mantle of Lo Encañado Valley with drainage classes for each geomorphic unit. The floodplain is represented by 3 geomorphic units being a very poorly drained Fen, poorly drained Meadow and somewhat poorly drained Meadow. Hillslopes are represented by colluvial deposits facing south and north. Each position occupied in the landscape represents according to the USDA (2012) [16] a different drainage class which is referred to the frequency and duration of wet periods under conditions similar to those which the soil developed. Drainage class numbers go in a scale from number representing the less well drained (or most saturated soils) up to number 6 representing the excessively drained soils (LE01: poorly drained, LE02: somewhat poorly drained, LE03: somewhat excessively drained, LE04: well drained, LE05: very poorly drained). Arrows represent the position of the soil profiles at Lo Encañado. **B.** Vegetation present at each site with dominant species shown in each site as a colored box **C. **Soil profiles described and sampled at each site. Numbers (H1, H2...) represent a pedogenetic horizon, which are distinguishable among them as they express different morphological properties which are defined in the Field. As pedogenetic horizons are not fixed depth, a scale of 1m is shown for each profile. Violet dashed line with asterisk represents the limit between topsoil and subsoil according to soil bacterial community clustering

Our study aimed to determine changes in the overall bacterial community structure across different types of mountainous soils and their distinct layers (horizons) occurring in a soil depth profile. This was carried out by describing and sampling soil profiles in five sites, reflecting five different drainage classes at four contrasting geomorphic positions: north and south facing colluvial slopes, floodplain marsh (fen) and floodplain meadow from a Mediterranean mountain environment in the central Andes (Lo Encañado floodplain) (Figure 1A). We hypothesized that soil bacterial community structure and putative ecological interactions among bacterial populations will vary among profiles given their distinctive drainage conditions. These differences will be accentuated at greater depths since other factors that also impact microbial composition besides water (such as temperature fluctuation, wind, plants and radiation), are only part of the surface environments but similar in all soil profiles given their proximity.

## Results

### General description of the sampling area: characterization of soil profiles and horizons

The sampling sites were located in Lo Encañado valley at around 2,490 meters above sea level (m a.s.l.), near a small (38.8 km^2^) tributary catchment of the Maipo River that drains towards a small oligotrophic and monomictic lake of the same name (Figure 1A, Additional file 1: Figure S1 and Additional file 2: Figure S2). The valley has been a priority area for the supply of drinking water for the city of Santiago since the 19^th^ century due to its high level of preservation and low human intervention.

The soil sampling was carried out in September 2019 at five soil profiles located at the bottom of the valley (Figure 1B). The selected soils exhibited contrasting properties and processes over a short distance (< 1.5 km), which are reflected here in five different classifications at the sub-group level (Table 1), according to soil taxonomy [15]. These differences are also observed based on five drainage classes according to the USDA, which consider the frequency and duration of wet periods under similar conditions to those under which the soil developed [16]. Regarding geomorphic position, the colluvium profiles LE03 and LE04 show differences in their properties and development, while both have organic (Oe) and mollic (A) horizons, their thickness changes (Additional file 6: Table S2). Pedogenic horizon characteristics changed between both profiles; while LE04 presents a thin cambic horizon (Bw) with weak subangular blocky structure, LE03 presents a slight reaction to HCl at 112 cm and a calcic horizon (Ck_1_ and Ck_2_) at the bottom, between 135 and 190 cm (Additional file 6: Table S1), reflecting drier drainage conditions. Other differences were observed between LE03 and LE04 included moisture, colour, structure (Additional file 6: Table S2), and physico-chemical properties (Additional file 6: Table S1). The meadow (fluvial) soils show slight pedogenic differences between LE01 and LE02, as both present cambic (Bw), mollic, and a thin organic horizon at the surface (Oi), while LE01 shows redox features in subsurface horizons (Bg). The most distinctive soil was LE05, a non-mineral (organic-fluvial) soil which is a histosol with fibric (Oi) and hemic materials (Oe), with sulfidic materials at the bottom of the sequence, reflecting a permanent water saturated conditions at that depth (Oese) (Additional file 6: Table S2).

**Table 1:** Main characteristics of soil profiles and their environment

Profile	Soil taxonomy (Great group)	Dominant plant species	Slope (°)	Geomorphic position	USDA soil drainage class
LE01	Cumulic Haploxeroll	*Juncus s*p.	0.86	Meadow (Fluvial)	2. Poorly drained
		*Acaena magellanica* (Lam.) Vahl *Acaena pinnatifida* Ruiz & Pav.			
LE02	Oxiaquic Haploxeroll	*Acaena magellanica* Ruiz & Pav.	2.29	Meadow (Fluvial)	3. Somewhat poorly drained
		*Juncus bufonius* L.			
		*Poa s*p.			
		*Taraxacum officinale* F.H. Wigg			
LE03	Typic Calcixeroll	*Ephedra chilensis* C. Presl	20.81	North facing colluvial hillslope	6. Somewhat excessively drained
LE04	Typic Haploxeroll	*Bromus berteroanus* Colla	34.22	South facing colluvial hillslope	5. Well drained
		*Haplopappus s*p.			
		*Vicia sp.*			
LE05	Typic Sulfisaprist	*Poa acinaciphylla* E. Desv.	0.57	Fen (Organic-Fluvial)	1. Very poorly drained

### Diversity of soil bacterial community structure in the soil profiles and horizons

The 16SrRNA amplicon sequencing of the different soil profiles yielded a total of 4,036,923 sequences. After rarefaction, we obtained 40,688 ASVs from 113 soil samples. Then, we applied filters for abundance (ASVs with more than 0.01% of relative abundance) and consistency (ASVs identified in at least two out of the three replicates), retaining 18,427 ASVs for posterior analyses. These ASVs encompassed 55 phyla and 695 genera across all soil profiles (Additional file 6: Table S3). The LE01 profile had the highest number of ASVs (n=4,324) and was associated with 40 phyla and 376 genera, followed with progressively fewer ASVs by LE02 (n=4,172 ASVs), LE05 (n=3,801 ASVs), LE03 (n=3,620 ASVs), and LE04 (n=3,477 ASVs).

**Fig. 2:**
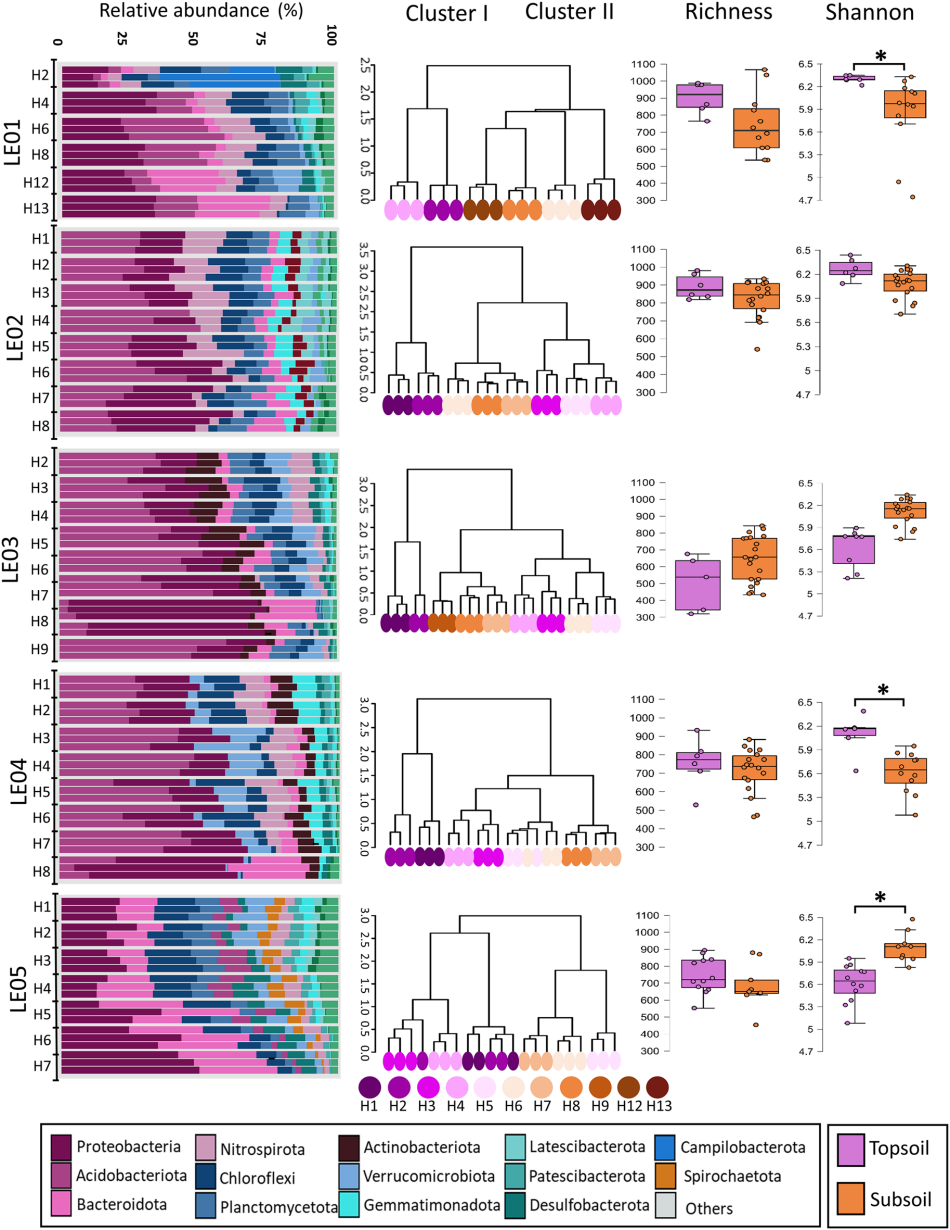
Soil bacterial community composition in five profiles along soil depth. The left vertical panel shows relative abundance of principal bacterial phyla, the middle panel shows Bray-Curtis dissimilarity analysis and the right vertical panel shows richness (Average numbers of OTUs) and diversity (Shannon index) along depths in the five soil profiles. The bottom and top of a box are the 25th and 75th quartiles, the horizontal line within a box is the median, and the ends of the whiskers are the limits of the distribution as inferred from the upper and lower quartiles. Dots are samples. *Asterisk indicate significance with Krustall-Wallis pairwise composition (topsoil-subsoil) (p<0.05)

The analysis of the taxonomic composition showed that in four of the five profiles, Proteobacteria and Acidobacteriota phyla predominated. The exception corresponds to the LE05 profile, where Bacteroidota and Chloroflexi accompany Proteobacteria as the most abundant (Figure 2, left panel and Additional file 6: Table S4). However, high variability in the taxonomic composition between profiles and horizons within a profile was observed (Figure 2 left panel and Additional file 6: Table S4). The relative abundance of Proteobacteria tended to decrease in deeper horizons in almost all the profiles, except for LE01. In LE01, we can highlight a drastic decrease in the relative abundance of Acidobacteriota in the deepest horizon (H13), where it represented only 4% of the total relative abundance, being more than 8% in the other horizons in the same profile. The opposite tendency of this phylum was observed in LE03 and LE04 profiles. Finally, Bacteroidota tended to decrease with depth in all profiles.

To assess whether bacterial composition differed between the shallowest horizons compared to the deepest ones in each profile, we performed a hierarchical clustering analysis, using Bray-Curtis dissimilarity indices, (Figure 2 middle panel). We observed that, for all profiles, the samples were separated into two major clusters: one which contained the samples from the shallowest horizon(s), and another containing mainly samples from the deepest horizons. Based on these results, we separated the samples into topsoil samples (TS), which were composed of the horizons that grouped shallow horizons from each profile, and subsoil samples (SS), which compiled the horizons grouped in the cluster formed by the deeper horizons from each profile. The horizons included in the TS corresponded to Oi (n=9), A (n=8), B1 (n=2), Oe (n=12), B (n=6), most of which were not present in the SS horizons. In terms of depth, horizons classified as TS reached a maximum depth of 52, 59, 33, 8 and 23 cm at sites LE01 to LE05, respectively (Additional file 6: Table S2).

To evaluate whether there were differences in alpha diversity between these two clusters of soil microbial communities, we performed a pairwise Kruskal-Wallis test. We found that richness did not change among the different profiles (Figure 2 right panel). On the other hand, alpha diversity evaluated by Shannon index of profiles LE01 and LE04 showed significantly higher diversity in their topsoil samples compared to their subsoil samples, while LE05 profile showed the opposite trend. Comparisons of the richness and alpha diversity between all samples showed significantly higher values (q-value ≤0.05 Kruskal-Wallis test) in TS than in SS samples (Additional file 3: Figure S3). In addition, comparisons of richness among profiles indicated that LE02 exhibited the highest richness. In terms of alpha diversity, both LE01 and LE02 showed the highest alpha diversity levels while LE05 profile showed the lowest (Additional file 4:Figure S4 and Additional file 6: Table S5). Furthermore, no significant changes in richness and alpha diversity were observed among the horizons within each profile (Additional file 6: Table S5).

To identify the shared ASVs between TS and SS samples across different sites, we employed a Venn diagram visualization. The results revealed that eight ASVs were common to all TS samples. Among these, five ASVs were classified under Proteobacteria, two under Nitrospira, and one under Fibrobacterota. On the other hand, we found that five ASVs were common to all SS samples, with three belonging to Nitrospira, one to Chloroflexi, and one to Planctomycetota.

### Co-occurrence networks from topsoil and subsoil samples

**Fig. 3:**
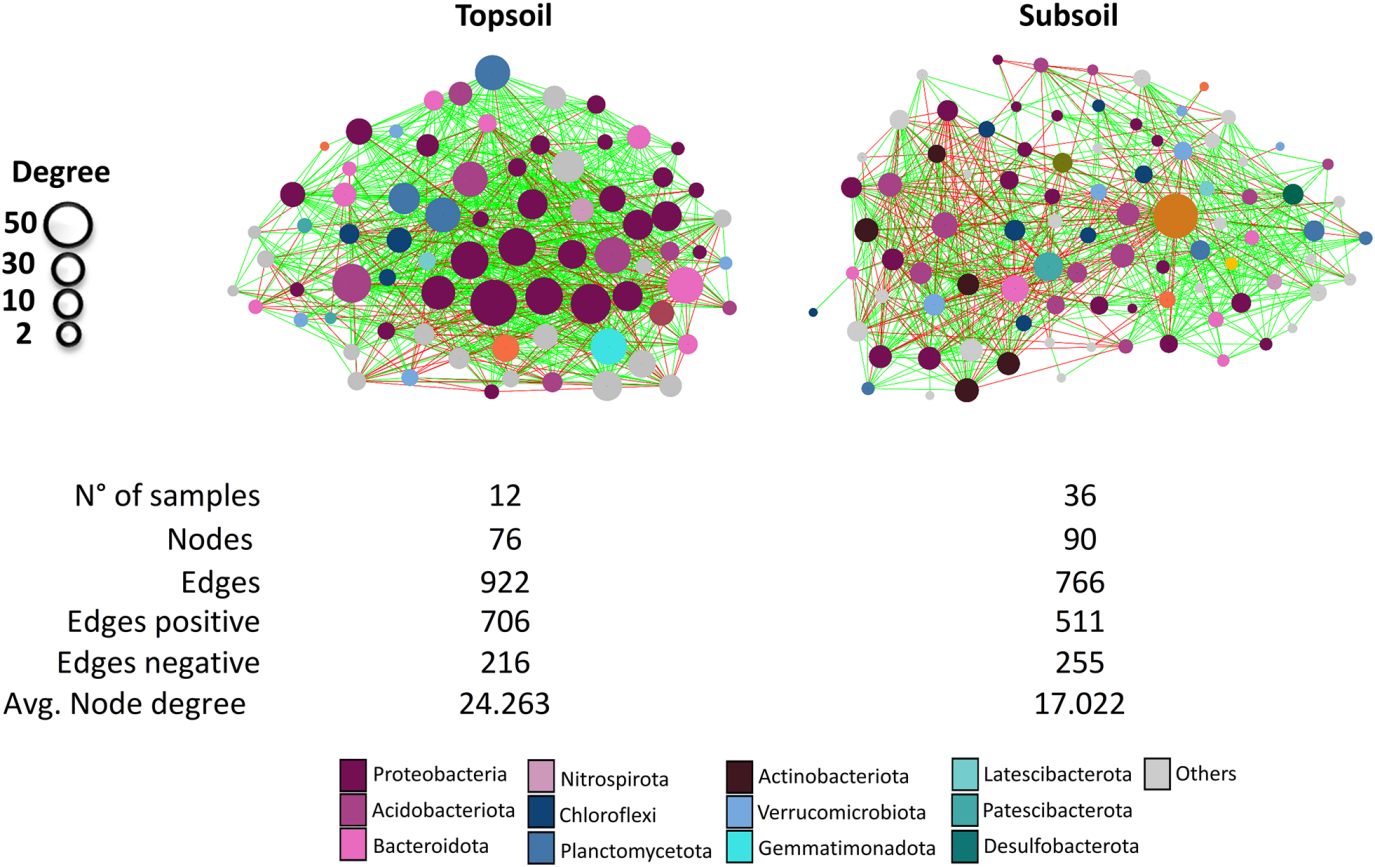
Co-occurrence networks. Complete bacterial co-occurrence networks of the TS (left panel) and SS samples (rigth panel). Interactions were inferred from bacterial ASVs abundances collapsed at the genus level. Each node represents a genus, and each edge represents a significant pairwise association between them (green lines: positive edges; red lines: negative edges). The different colors of nodes represent distinct phyla. Node sizes are proportional to the number of connections (degree) of each network (maximum node degree was 50 and 45 for TS and SS network respectively)

To further analyze the differences in the bacterial communities associated with TS or SS samples, two co-occurrence networks were constructed (Figure 3). Both networks were mostly composed of ASVs belonging to the phyla Proteobacteria, Acidobacteriota, Actinobacteriota, and Bacteroidota. The TS network exhibited a higher number of edges (mainly positive edges) and average node degree than the SS network (Figure 3, lower panel). Overall, these results revealed differences in bacterial community structure and co-ocurrence and mutual exclusion patterns between the TS and SS communities.

### Differentially abundant bacterial taxa between topsoil and subsoil samples

**Fig. 4:**
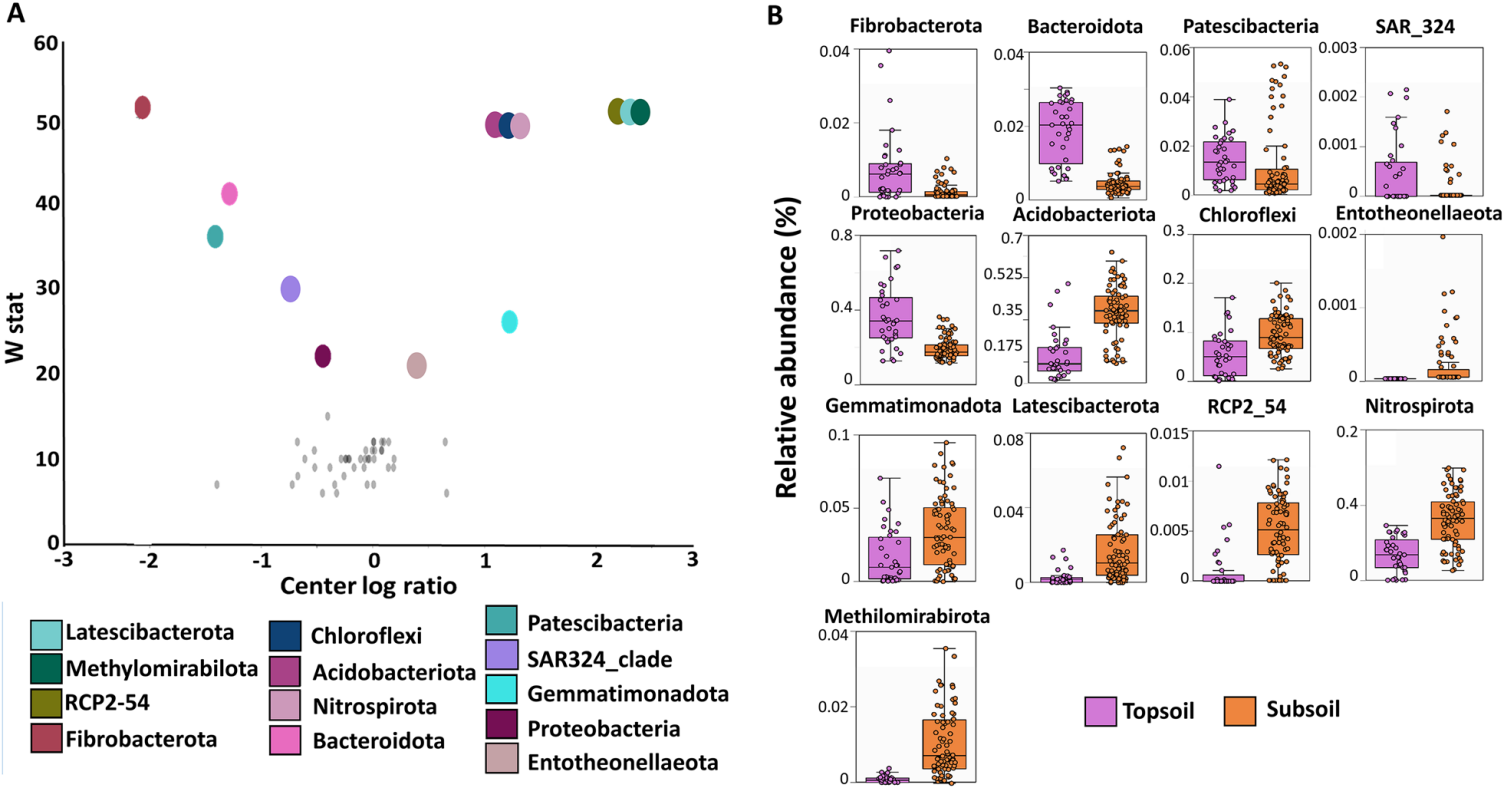
Differential abundance analysis. **A**. Differentially abundant microbial phylum identified by ANCOM. Volcano plot of differential abundance at the group level (topsoil and subsoil), clr are represented on the x-axis and W-statistics on the y-axis. The crl (center log ratio) is a measure of the effect size difference for a particular species between the study groups, and the W-statistic represents the number of times of the null-hypothesis (the average abundance of a given phylum in a group is equal to that in the other group) was rejected for a given phylum. *p*-values with good control of the Benjamini-Hochberg correction (FDR) at 5% type I error rate, are already embedded in the ANCOM test before the final significance based on the empirical distribution of a count random variable called W. **B.** Box plot comparing relative abundances between topsoil samples (violet) and subsoil samples (orange) of the phyla identified as differentially abundant. The bottom and top of a box are the 25th and 75th quartiles, the horizontal line within a box is the median, and the ends of the whiskers are the limits of the distribution as inferred from the upper and lower quartiles. Dots are samples. Note that significance of phyla among groups was tested using three approaches. First we performed an ANCOM test, followed by a metagenomeSeq analysis and after a more conservative univariate analysis using Mann-Whitney U test (p < 0.05). *Asterisk the phyla that were considered enriched were those that were significantly enriched in at least two of the three (Additional file 6: Table S6)

To identify which bacterial phyla and families contributed to the TS and SS differences in the community composition of the Lo Encañado sites, we performed an analysis of the composition of the microbiome (ANCOM) followed by a MetagenomeSeq analysis and Mann–Whitney U test by profile (Figure 4, Additional file 6: Table S6 and Additional file 7: Table S7). From this analysis, we observed that several phyla were enriched in the TS or SS soil samples in the different profiles, indicating that the distinct soil properties of these two soil layers could shape their respective microbial community structure. For instance, we found that Bacteroidota was enriched in the TS samples from all sites and the same trend was found for several families belonging to this phylum. The Flavobacteriaceae family was found enriched in all TS sites, and the Chitinophagaceae and Sphingobacteriaceae families were detected enriched in TS in four of the five sites (Additional file 6: Table S6 and Additional file 7: Table S7). Additionally, we found that Proteobacteria were enriched in the TS soils of LE02, LE03 and LE04. In this phylum, we identified that families Acetobacteraceae, Caulobacteraceae, Pseudomonadaceae, R7C24 and Sphingomonadaceae where enriched in at least three of the five sites. Fibrobacterota and Abditibacteriota was enriched in the TS of two profiles (LE01 and LE02 for Fibrobacterota and LE02 and LE03 for Abditibacteriota). On the other hand, Chloroflexi and Nitrospirota were enriched in the SS samples of almost all profiles, except for LE05. In the phylum Chloroflexi, the families S085, Gitt-GS-136 and P2-11E were detected in three of the four sites and in Nitrospirota two families were dominants in SS samples (Leptospirillaceae and Nitrospiraceae) (Additional file 6: Table S6 and Additional file 7: Table S7).

When comparing all profiles in one ANCOM, thirteen phyla exhibited significant differences in abundance between TS and SS samples (Figure 4) (the phyla considered differentially abundant were those that were significantly enriched in at least two out of the three conducted tests)**.** From these, five belonged to TS samples (Fibrobacteres, Bacteroidetes, Patescibacteria, SAR_325 and Proteobacteria) and eight to SS samples (Acidobacteriota, Chloroflexi, Entotheonellaeota, Gemmatimonadota, Latescibacterota, Methylomirabirota, Nitrospirota and RCP2_54).

## Discussion

### Landscape position, soil distribution, and soil features

In this study, we examined the soil bacterial community structure across five closely located soils distributed within contrasting geomorphic positions from an Andean valley (Lo Encañado), located in the lower alpine vegetation belt with a cold summer mediterranean climate condition (Csc under Köppen climate classification) [17]. Our aim was to assess the changes in taxonomic composition, diversity, and co-occurrence networks of the bacterial community among soil types, drainage classes, and depth.

The Lo Encañado valley was carved during the Pleistocene glaciations and subsequently filled with fluvial deposits, while the valley sides are covered with a continuum of colluvial deposits. The soils found at the valley reflect a high variety of properties and associated pedogenic processes, similar to other alpine regions of the world in terms of the high diversity of soils that could be found in small areas [2]. Small changes in landscape position produce changes in the frequency and duration of wet periods, affecting the conditions under which the soils are develop and these differences are defined under the concept of drainage class by the United States Department of Agriculture [16]. Four of the five examined sites correspond to mineral soils (LE01, LE02, LE03 and LE04) and one site corresponds to an organic soil (LE05) as defined by soil taxonomy [15]. An interesting result was the presence of a Mollic horizon in all the mineral soils, reflecting the connection between these soils and the soils found at the pre-Andean ranges and lowlands of the Maipo river basin [18]. This reflects the prevalence of similar soil forming factors at least within this vegetation belt. In this sense, we can point out the dominance of Mediterranean climate conditions which allow the formation of Mollisols within the lower Andean belt.

The contrast of drainage conditions could mostly be explained by the geomorphic position of the soil profiles, namely the topography soil forming factor described by Jenny, [19]. Additionally, in Lo Encañado valley, the differences between the selected sites are enhanced as the soils are on relatively stable surfaces and therefore have been exposed to the environment for a significant period of time, experiencing pedogenic processes that are reflected in their properties. For instance, the presence of a subangular blocky structures in all mineral soils is a reflection of the prolonged action of pedogenic processes related to vertical and lateral shrinking forces common in soils that experience fast drying, which can be seen increased in Mediterranean climates as fast drying cycles occur every year during summer [20, 21]. As reported in many studies, soil structure has a great influence on microorganisms due to its diverse niches and gradients of water, gases, and nutrients [22]. In this sense, soil features reflect both the intensity of the processes that occurred and the differences between the profiles resulting from pedogenetic development.

### Effects of depth and soil profiles on bacterial community structure

While the effect of geographic distance has been proposed as a factor influencing soil microbial communities [23], there seems to be a greater consensus that variations of soil properties (i.e. pH, nutrients, carbon content, salinity, texture) are decisive factors in shaping bacterial community structures [11, 24–26]. This can be seen clearly at our study site, where, despite the proximity of the selected profiles, bacterial community structures show significant differences in their structure, suggesting that the selection of soils from contrasting geomorphic positions and drainage conditions represented distinct soil environments. However, as it has been reported for other soil environmental variables [11], the effects of geomorphic positions and drainage conditions on soil microbiome appears to be context-dependent.

Between soil profiles and horizons, there are patterns of diversity that form a clear structure in the soil microbial community. This is observed when analyzing the replicates for the soil samples analyzed here, where there is a relationship of microbial diversity and abundance that remained very similar, which shows the benefit of a sampling strategy based on pedogenic horizons. It should be considered that there are biotic and abiotic factors that strongly influence the assembly of a microbial community, both at the level of diversity and abundance. For instance, the effect of soil moisture produced a gradient of richness and diversity (from higher to lower) from humid, subhumid to semiarid soils [27]. However, studies have established these differences as dual parameters, considering for example that variations in ultraviolet (UV) radiation do not alter diversity and richness indices in bacterial communities at the genus level, but a decrease in water resources associated with precipitation generates inversely proportional changes in their diversity and richness (low abundance and higher diversity) [28]. Moreover, other authors relate this effect to thermal and hydric factors, which, combined, trigger a rapid decrease in bacterial diversity in soils, either in the short term [29] or in the long term [30], associating their results to a rapid response to adverse climatic conditions.

Our data indicated that soil classified as TS, which corresponded to the most superficial horizons, were mostly shaped by different soil types in comparison to SS horizons. Both features (depth and soil type) have been used to classify TS and SS samples in studies carried out in several soil types and geographic locations [9, 10, 31]. However, differences on composition of soil bacterial communities and their abundance tend to locate the edge of the TS-SS transition at different depths among the five analyzed profiles. This might be related to the fact that TS horizons were associated with different dominant plant species, which is a biotic effect that could shape the composition of soil microbiomes since root exudates have been considered main drivers in shaping rhizosphere microbial communities [32, 33]. Plant root exudates provide photosynthate carbon for microbial growth [34], while in a bidirectional relationship, rhizosphere microorganisms also contribute to plant nutrition and plant resistance to biotic and abiotic stresses [35]. For example, taxonomic analysis revealed an abrupt change between the rhizosphere and the rest of the non-rhizospheric soils of Chilean altiplanic native plants from the Andean grasslands soils [36]. In addition analysis of 21 native plant species organized into three vegetation belts in the Andes in the Atacama Desert showed that plants appears to determine plant-specific microbial community composition in the soil associated to root system and showed that bacterial communities modify their ecological interactions, in particular, their positive:negative connection ratios in the presence of plant roots [37]. Thus, spatial differentiation of TS and SS as environments must be interpreted with caution, especially in studies of bacterial community assembly processes along a soil depth gradient. On the other hand, microbial community structure, as evaluated by the Bray-Curtis dissimilarity analysis, showed a coherence between the microbial structures of the SS horizons of the same profile, suggesting that there is a link between additional environmental factors and microbial community structure [38]. For instance, changes in bacterial community composition associated to drainage processes have been reported [39, 40]. In particular, Conrad and coworkers (2020) [40], discussed the consequence of flooding and drainage processes in the context of oxygen sensitivity of microorganisms in the soil environment. The flooding and drainage factor turns out to be crucial for the presence of anaerobic microorganisms in soils with lower drainage capacity. Considering that soils exposed to permanent flooding events will have low oxygen diffusion, they contain strict anaerobes such as Firmicutes, while soils with good drainage capacity will tend to include individuals tolerant to oxygen and also tolerant to desiccation [40]. Supporting this hypothesis, we detected an increased relative abundance of Firmicutes members in a very poorly drained site (LE05). In addition, the lowest microbial diversity was also found in LE05 because this soil is saturated with water most part of the year and so, the anoxic conditions might prevail.

Alpha diversity was also lower for the deeper horizons (SS samples) of almost all profiles, a result that is displayed in many other studies using a variety of different techniques, like pyrosequencing [9, 41, 42], clone libraries [43], and fingerprinting approaches [44, 45]. Although we did not establish a direct relationship between the decrease in alpha diversity and a decrease in nutrients, this result may suggest that changes in soil environmental conditions with depth represent a strong ecological barrier, and that many surface living microorganisms are less likely to thrive in deeper soil environments. The fact that this trend is not evident in LE02 and LE03 in terms of diversity may be due to factors that cannot be clearly explained by the soil physico-chemical parameters described in the present study.

In terms of dominant taxa, Proteobacteria and Acidobacteriota were the most dominant taxa in all profiles, except in LE05, where Acidobacteriota is surpassed in relative abundance by Bacteroidota and Chloroflexi. The dominance of these phyla has been observed in a wide variety of soil types and depths in various ecosystems worldwide, indicating that they play a fundamental role in this type of ecosystems [36, 41, 46]. A higher proportion of Bacteroidota was observed in LE05 profile respect to the four other sites, as well as a decrease in Acidobacteriota accompanied by Chloroflexi. The higher abundance of Bacteroidota in the LE05 profile, which is an organic soil, could be explained by their copiotrophic life attributes [47].

Regarding changes in the bacterial distribution across the distinct layers in a soil depth profile we observed that the structure of the bacterial community displayed a specific stratification between TS and SS. At phylum taxonomic level, we found several phyla that were enriched or decreased in the SS with respect to the TS. These results provide valuable information for microbial ecology of deep soils, which remains particularly limited, especially in mountain pristine environments from Mediterranean regions. Members of Proteobacteria and Bacteroidota have shown to be dominant in TS horizons and to decrease from the surface to deeper horizons [41, 42, 48], comparable to our results where Bacteroidota was enriched in all TS horizons and Proteobacteria was enriched in the TS in LE02, LE03, and LE04 (Table S6). It is possible that the enriched abundance of the phylum Proteobacteria in TS could be due to their adaptation to the high C input from root exudates [47, 49, 50]. As has been reported in other studies [9, 26, 51], we found that Chloroflexi was enriched in the SS samples. Chloroflexi are facultative anaerobic bacteria that include autotrophic, heterotrophic, and mixotrophic taxa [52, 53]. It has been reported that Chloroflexi has adapted to grow in conditions of low nutrient concentrations, which may explain their enrichment in the SS samples [54, 55] and their decrease in fertilized soils [56]. Therefore, due to their diverse “life strategies”, different physiological strategies may be responsible for their coping with the hostile subsurface environments.

Our results on bacterial diversity and composition support the idea that depth generates environmental gradients that affect soil bacterial composition and structure [38, 41, 57]. To investigate whether co-occurrence and mutual exclusion patterns are also affected by depth, we generated co-occurrence networks of TS and SS samples. Overall, bacterial co-occurrence networks showed that, in comparison with SS, the TS network exhibited a greater number of edges (mainly positive), and a higher average node degree. In the TS horizons, the presence of roots could increase the amount of root exudates, which could influence community structure by either stimulating or repressing bacterial growth [58] or by altering the soil microhabitat [59, 60]. In contrast, a lower presence of roots in the SS horizons could be associated with a reduction of available nutrients due to the reduction of inputs deposited by roots. Therefore, our network analysis matches other studies that have shown that a higher availability of nutrients is associated with the presence of more connected soil bacterial networks [61, 62]. Also other possibilities could be considered to explain this network structure, for example, that microbial communities with more complex and connected co-occurrence networks are considered to be more resilient to environmental stresses than those with simpler networks [63]. This would agree with our study since TS communities, given their superficial position, are more subjected to environmental and climatic changes than communities in SS soils. However, further studies should be conducted to clarify the drivers of changes in co-occurrence networks among TS-SS bacterial communities.

## Conclusion

Soils in the lower alpine vegetation belt locality of Lo Encañado show a high pedogenic variability which is closely linked to its geomorphological position in the landscape and is reflected in the drainage conditions described. This spatial pattern of soil variation is connected in a different way to the microbial communities that each soil profile hosts, as their structure is closely linked in all subsoil or topsoil horizons of the same soil profile. The spatial pattern (SS versus TS) of the soil microbial communities could not be clearly linked to soil physico-chemical properties or depth, but the similarity trends suggest that moisture availability might play an important role, as different soil developments could create long-term conditions for niche differentiation and the creation of particular microbial community patterns for each site. In fact, more bacterial members interacted within topsoils versus subsoils, showing also higher average connectivity. The composition of soil bacterial communities and the abundances of specific taxa could be considered to identify the transition from topsoil to subsoil horizons, like Fibrobacterota and Bacteroidota for shallower soils and Chloroflexi, Latescibacterota and Nitrospirota for deeper soils. The close relationship among topsoil microbial communities, despite differences in landscape position and vegetation cover, suggests that their structure is mainly influenced by shared soil surface environmental variables (like temperature fluctuations, wind, plants, and radiation) given their geographic proximity. Finally, we conclude that a geomorphological and pedological approach is an adequate method to capture the microbial biodiversity in a mountain environment.

## Materials and methods

### Site description

The sampling area is located in the Lo Encañado valley (33°40’ S, 70°08’ W) at ~2500 m a.s.l. in the Andes mountains in the central region of Chile (Figure 1 and Additional file 1: Figure S1). The catchment is composed of Oligocene to Miocene volcanic sedimentary sequences (basaltic to dacitic lavas, epiclastic and pyroclastic rocks) belonging to the Abanico and Farellones formations, together with granite and quartz monzonite plutons of Miocene age [64]. The valley bottom consists of a typical mountain through a valley which was carved during the Pleistocene glaciations and subsequently filled with fluvial deposits, while the valley sides are covered with a continuum of colluvial deposits. The climate in this section of the Andes is described as semi-arid with a pronounced winter precipitation maximum and large interannual variability in precipitation and streamflow; precipitation mainly occurs as snow during winter, while in summer, flash hail or snow hailstorms at higher elevations may occur [65, 66]. According to data reported by Bodin et al. [67], the nearest weather station is the Embalse del Yeso (2475 m a.s.l., 33°40’ S, 70°05’ W), which for the period 1962-1991 reported a mean annual air temperature of 8.3 °C, with monthly temperatures ranging from 1.6 °C in July to 14.3 °C in January. The Embalse del Yeso station recorded a mean annual precipitation (MAP) sum of 524 mm water equivalent (s.d. 322), with extremes in January of 4 mm and 124 mm in July, evidencing high interannual and annual variability, January being the driest month (~ 4mm) and July the wettest (~120 mm) [67]. The soil thermal regime of a location in the nearby Laguna Negra catchment was measured by Bodin et al. (2010) [67] at different altitudes. For the site at 2475 m.a.s.l., mean annual soil temperatures of 8.9 °C (s.d. 4.5), 9.6 °C (s.d. 6.9), 8.9 °C (s.d. 5.4) and 8.4 °C (s.d. 4.0) were recorded at 0 cm, -10 cm, -50 cm, and -100 cm, respectively. Their data record shows that soil temperature below 20 cm did not reach a freezing point during their measurement period at this altitude. According to this data, the soil temperature regime would be frigid following soil taxonomy nomenclature [15].

In this area, the western flank of the Andes has four vegetation belts [68–70]. Lo Encañado catchment is entirely located above the treeline with 35% of the area occupied by the alpine dwarf scrub belt (lower alpine zone), where this study is located (Additional file 1: Figure S1B).

### Soil sampling

Five soil profiles located at similar altitudinal levels (~2500 m a.s.l.) were examined in spring 2019 (Figure 1). These profiles are representative of the mosaic of geomorphic positions that can be found in a typical alpine trough valley in the lower alpine vegetation belt alpine dwarf scrub according to Luebert and Pliscoff [68]. For each site, dominant plant species were collected and later identified using taxonomic keys. The selection of the profiles took the five major soil forming factors into account (climate, organisms, parent material, time) [18] to keep all factors as constant as possible, except topography. Based on the extent of glaciation in the area, the landforms are considered to be post Last Glacial Maximum, estimated to have a maximum age of 35 thousand years [71]. All soils formed after sediments: three of them are located at the valley bottom in a flat surface and are considered to be meadow soils, while two of them are located in the opposite hillslopes of colluvial material. Soil features were identified and described using methods outlined in the USDA Soil Survey Manual [16]. Soil profiles are described according to the arrangement and properties of soil horizons, which correspond to layers parallel to the soil surface that can be distinguished from adjacent layers by a distinctive set of properties (e.g. color, moisture, texture, structure) produced by soil-forming processes [16]. Each soil profile corresponds to a different soil type and was classified at the subgroup level according to soil taxonomy [15].

To analyse soil microbes, each section was divided into three vertical sections and one sample was taken from each section (Additional file 5: Figure S5), resulting in three replicates for each horizon. In profiles LE01, LE02, and LE05, each section corresponded to a different face of the soil pit, while in profiles LE03 and LE04, the same face was divided into three sections. Section replicates were separated by at least 40 cm. Soil samples were extracted from each section using sterile tools for metabarcoding analysis. The samples were stored at −20 °C and transported to the laboratory where they were kept at the same temperature and processed a few days after sampling.

### Soil analysis

Soil samples for physical and chemical analysis were air-dried, gently crushed and sieved to obtain a <2mm fraction. The chemical variables analyzed in each soil sample were pH (aqueous solution, mineral sample 1:2,5 and organic sample 1:5), electrical conductivity (determination by conductimetry, mineral samples by saturation extract and organic samples by 1:5 extract), soil organic matter (SOM) (calcination method), micronutrients (Copper “Cu”, Iron “Fe”, Manganese “Mn”, Zinc “Zn”) (DTPA extraction), available nitrogen (using Kjeldahl distillation), available phosphorus (using a P-Olsen method) and available potassium (extraction with 1mol/L ammonium acetate solution at pH 7.0 and subsequent determination by atomic absorption and emission spectrophotometry), based on the methods described by Sadzawka et al. (2004) [72]. The analyzed physical variables were texture (Bouyoucos), bulk density (cylinder method) and soil water retention curve, based on the methods described by Sandoval et al. [73].

### Microbial community analysis

To increase the chances of obtaining DNA from soil samples, we followed the protocol described in Mandakovic et al. [74]. Briefly, we combined an incubation of 5mL of lysis buffer [100 mM Tris-HCl; pH 8, 100mM Na EDTA; pH 8, 100mM Na_2_HPO_4_, 1.5M NaCl, 1% (w/v) CTAB] with five grams of soil and mixed using a vortex followed by incubation at 65 °C for 2 hours with mixing every 30 minutes. Subsequently, the mixture was centrifuged at 9000g for 5 min at room temperature and the supernatant was transferred in a clean tube and added 0.5 volume of ethanol 100%. In the next step, silica columns from the DNeasy Blood & Tissue kit (Qiagen) (Hilden, Germany) were used to capture DNA, following the manufacturer's recommendations from that step. The integrity of the DNA was evaluated through electrophoresis with 1% agarose gel, DNA quantification was performed through fluorometry using Qubit (Invitrogen) and DNA was stored at 4 °C until further procedures. Microbial DNA was amplified using a bacteria-specific primer set, 28 F (5′-GA GTT TGA TCM TGG CTC AG-3′) and 519R (5′-GWA TTA CCG CGG CKG CTG-3′), flanking variable regions V1-V3 of the 16S rRNA gene [75, 76]. Sequencing was performed by Mr. DNA (Shallowater, TX, USA) using an Illumina MiSeq platform in an overlapping 2×300bp configuration with a minimum throughput of 20,000 reads per sample [4]. The Fastq processor application on the website www.mrdnafreesoftware.com created the file formats expected by QIIME 2 for downstream analysis.

Bioinformatic analysis was performed with QIIME 2 2021.11 [77]. Raw sequence data was demultiplexed and quality filtered using the q2‐demux plugin followed by denoising with DADA2 [78] (via q2‐dada2). After denoising process we obtain 17,154,520 Sequences. Taxonomy was assigned to ASVs using the q2‐feature‐classifier classify‐sklearn naïve Bayes [79] against the SILVA v.138 97% database [80]. Libraries were filtered to remove low abundance and sparse ASVs (occurrence in at least two out of three replicates or at a total relative abundance of > 0.01% of all sequences) to analyze data using representative ASVs from each site so we could make robust comparative analyses between the different bacterial communities.

All ASVs were aligned with Mafft [81] (via q2‐alignment) and used to construct a phylogeny with fasttree2 [82] (“via q2‐phylogeny” QIIME2 plugin). After rarefaction to 40.688 sequences per sample (subsampled without replacement), microbial alpha diversities were computed using observed_features and Shannon indices (“via qiime diversity core-metrics” QIIME2 plugin). Alpha diversity was compared between profiles and horizons using the Kruskal-Wallis test (“qiime diversity alpha-group-significance” QIIME2 plugin). P-values were corrected with a Benjamini and Hochberg correction method and false discovery rate (FDR) correction was applied as a multiple comparisons method. FDR < 0.05 was considered statistically significant. For beta diversities Bray Curtis distance matrices was calculated with (“via qiime diversity core-metrics” QIIME2 plugin). Ward’s hierarchical clustering method based on Euclidean distance was also applied using Microbiome Analyst software [83].

To identify differential abundant taxa, we performed an analysis of the composition of microbiomes (ANCOM) [84]. ANCOM considers the compositional nature of the dataset [85] and compares absolute abundance in the community (via “qiime composition ancom” Qiime2 plug-in) [84]. Furthermore, the significance of phyla among groups was tested using two additional approaches; first, we performed a MetagenomeSeq [86] analysis followed by a more conservative univariate analysis using the Mann-Whitney U test (p < 0.05). The resulting p-values were adjusted using Benjamini-Hochberg as an FDR correction method. Both analyses was performed using Microbiome Analyst software [83].

Bacterial co-occurrence networks from topsoil and subsoil samples were generated as described by Mandakovic et al. [74] using genus as nodes in the networks (ASVs were pooled from replicates and collapsed at the genus level) and as edges of positive or negative correlations. Significant co-presences or mutual exclusions across the samples were identified using the CoNet method [87] using a multiple ensemble correlation. Four similarity measures were calculated: Bray Curtis and Kullback-Leibler non-parametric dissimilarity indices, and Pearson and Spearman rank correlations. Distribution of all pairwise scores between genus was computed for all top soil or subsoil samples to enrich the network with genus nodes. For each measure and each edge, 100 renormalized permutations and bootstrap scores were generated according to Faust and Raes [88]. The networks were displayed by Cytoscape [89], which analyzed the statistics of the networks.

## Supplementary Information

**Additional file 1: Figure S1**. Location of the study sites. A. Location of the study site in the Central Andes of Chile. B. Lo Encañado Valley according to vegetation belts following distribution modelled by Luebert and Pliscoff. DEM: Digital Elevation Model.

**Additional file 2: Figure S2**. View toward Lo Encañado valley with the typical U shape of glacial origin. Bottom left is the lake of the same name; flat valley bottom consists of a flood plain sustaining an alpine meadow. The limit between the valley bottom and lateral escarpment are filled with colluvial deposits. Soil profiles are indicated by a white arrow.

**Additional file 3: Figure S3**. Alpha diversity and richness of TS and SS samples. Comparison of alpha diversity measures between the topsoil samplesand subsoil samples. Horizontal bars within boxes represent median. The tops and bottoms of boxes represent 75th and 25th quartiles, respectively. All outliers are plotted as individual points. * Asterisk denotes significant difference at the P ≤ 0.05 level using Kruskal-Wallistest.

**Additional file 4: Figure S4**. Alpha diversity and richness between different sites. Comparison of alpha diversity measures between the different sites. Horizontal bars within boxes represent median. The tops and bottoms of boxes represent 75th and 25th quartiles, respectively. All outliers are plotted as individual points. *Letters denotes significant difference at the P ≤ 0.05 level using Kruskal-Wallistest.

**Additional file 5: Figure S5.** Sampling strategy. Soil sampling procedure and nomenclature used for biological samples for a ficticious profile LEX.

**Additional file 6: Table S1.** Physicochemical and environmental measurements. **Table S2.** Selected properties of the Horizons at Lo Encañado. **Table S3.** Community metrics including phyla, genera and OTU richness between soil profiles. **Table S4.** Relative abundance of bacterial phyla among profiles and horizons. **Table S5.** Kruskal-Wallis test for alpha diversity analysis. **Table S6.** Differential abundance analysis between topsoil and subsoil samples at phylum level.

**Additional file 7: Table S7.** Differential abundance analysis between topsoil and subsoil samples at family level.

## Data Availability

The raw sequences were submitted to NCBI under BioProject: PRJNA805231.
