# Supplementary figures and images for: Topsoil and subsoil bacterial community assemblies across different drainage conditions in a mountain environment
Source: Biol Res. 2023 Jun 24;56:35. doi: 10.1186/s40659-023-00445-2 (PMC10290380; doi:10.1186/s40659-023-00445-2)

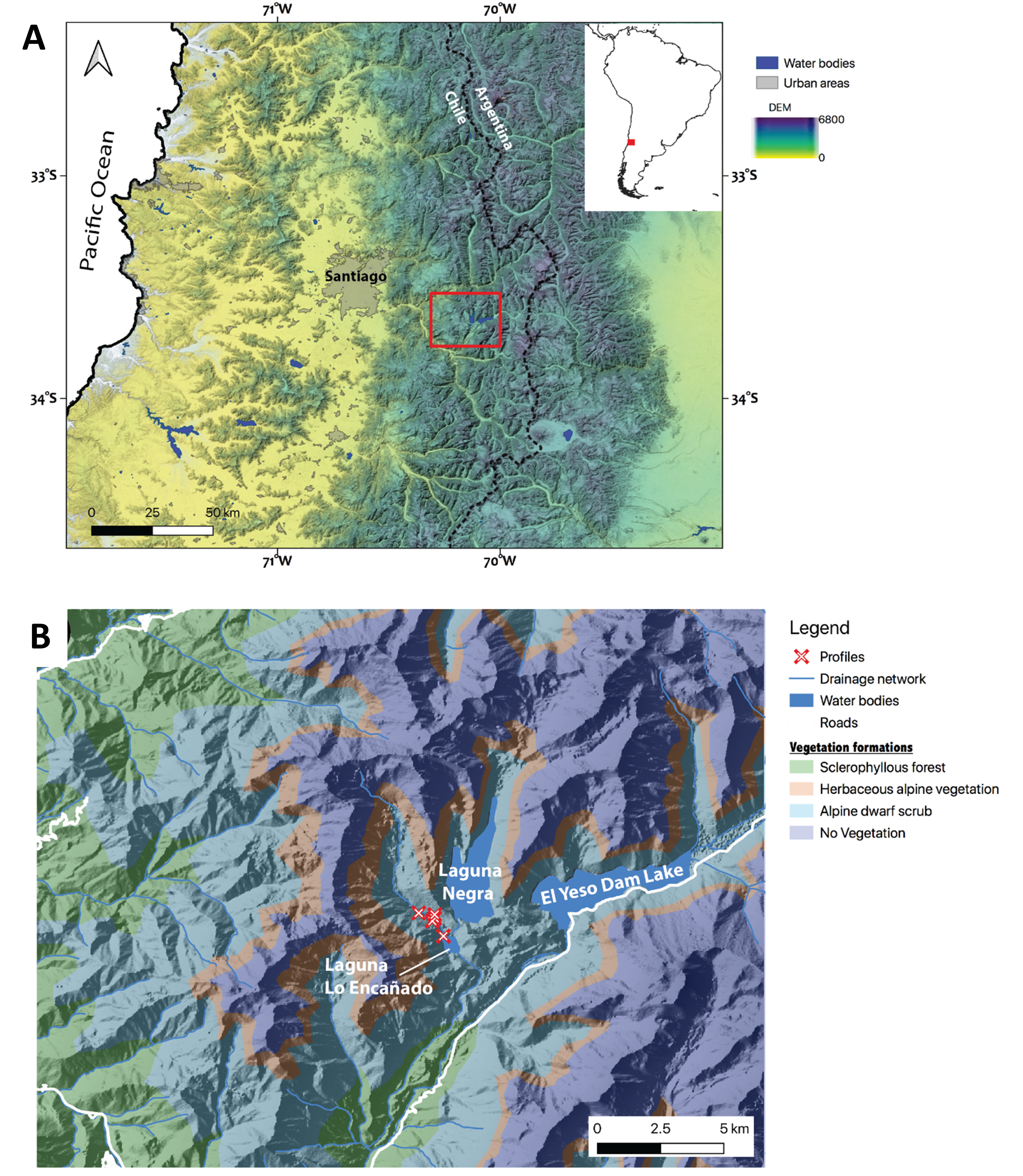

Supplement: Supplementary file 1 — Additional file 1: Figure S1. Location of the study sites. A. Location of the study site in the Central Andes of Chile. B. Lo Encañado Valley according to vegetation belts following distribution modelled by Luebert and Pliscoff. DEM: Digital Elevation Model. [file 40659_2023_445_MOESM1_ESM.tif]

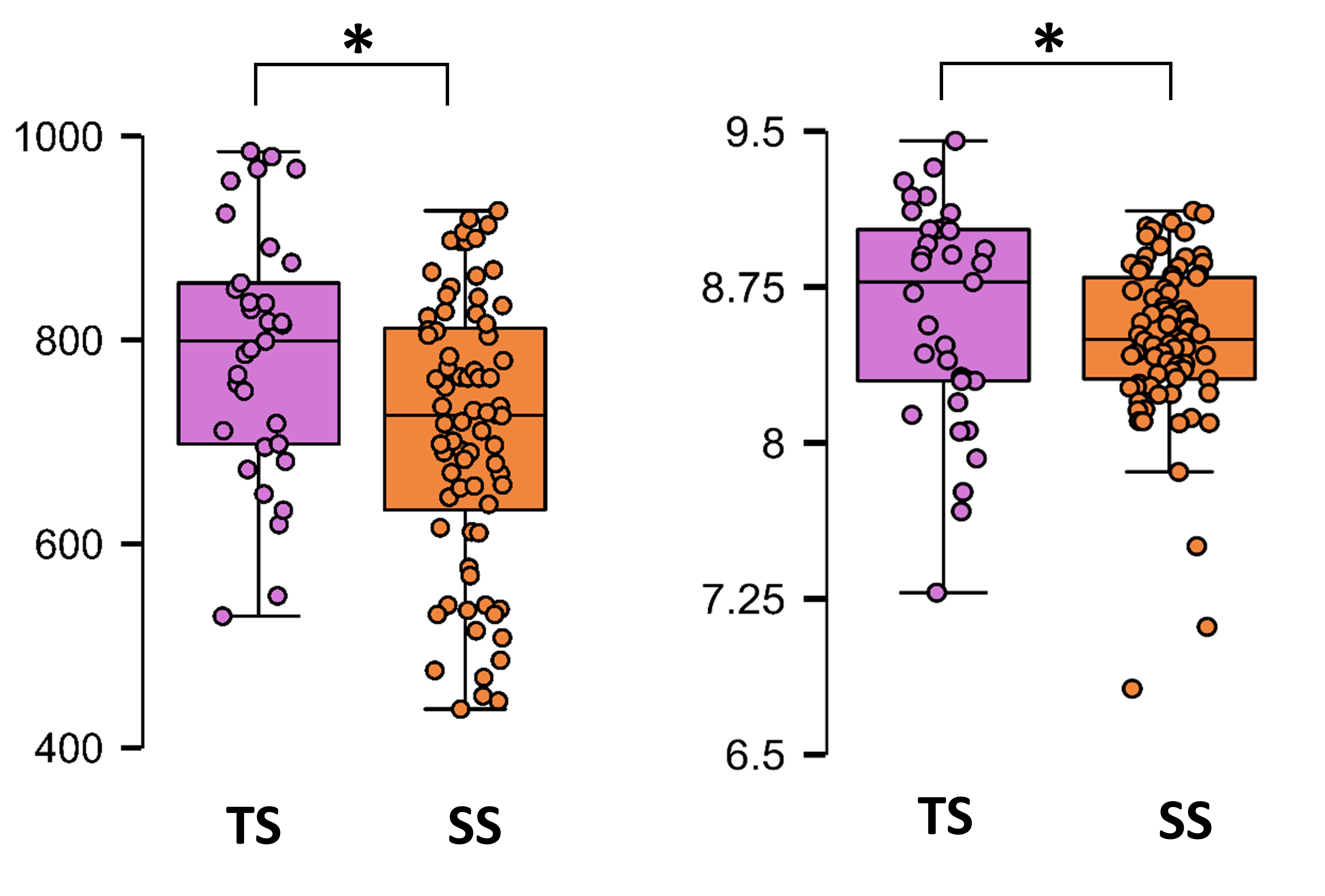

Supplement: Supplementary file 3 — Additional file 3: Figure S3. Alpha diversity and richness of TS and SS samples. Comparison of alpha diversity measures between the topsoil samplesand subsoil samples. Horizontal bars within boxes represent median. The tops and bottoms of boxes represent 75th and 25th quartiles, respectively. All outliers are plotted as individual points. * Asterisk denotes significant difference at the P ≤ 0.05 level using Kruskal-Wallistest. [file 40659_2023_445_MOESM3_ESM.tif]

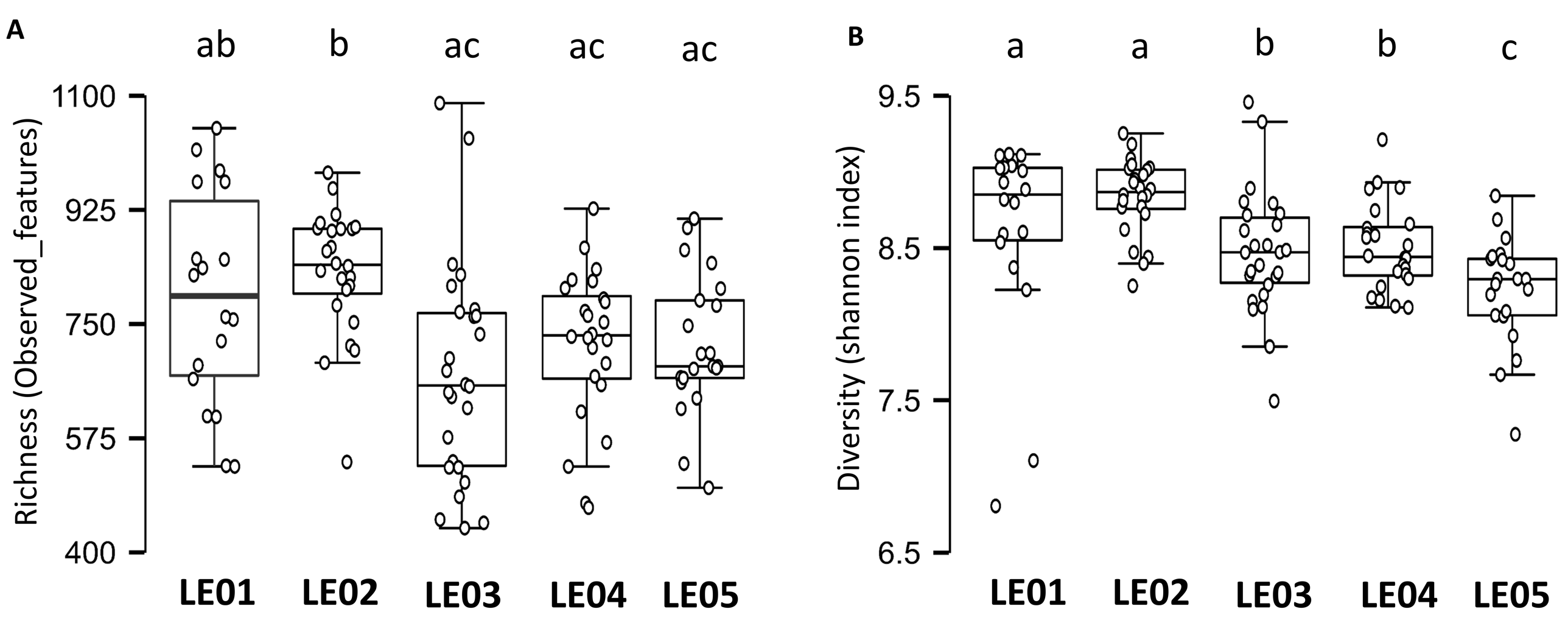

Supplement: Supplementary file 4 — Additional file 4: Figure S4. Alpha diversity and richness between different sites. Comparison of alpha diversity measures between the different sites. Horizontal bars within boxes represent median. The tops and bottoms of boxes represent 75th and 25th quartiles, respectively. All outliers are plotted as individual points. *Letters denotes significant difference at the P ≤ 0.05 level using Kruskal-Wallistest. [file 40659_2023_445_MOESM4_ESM.tif]

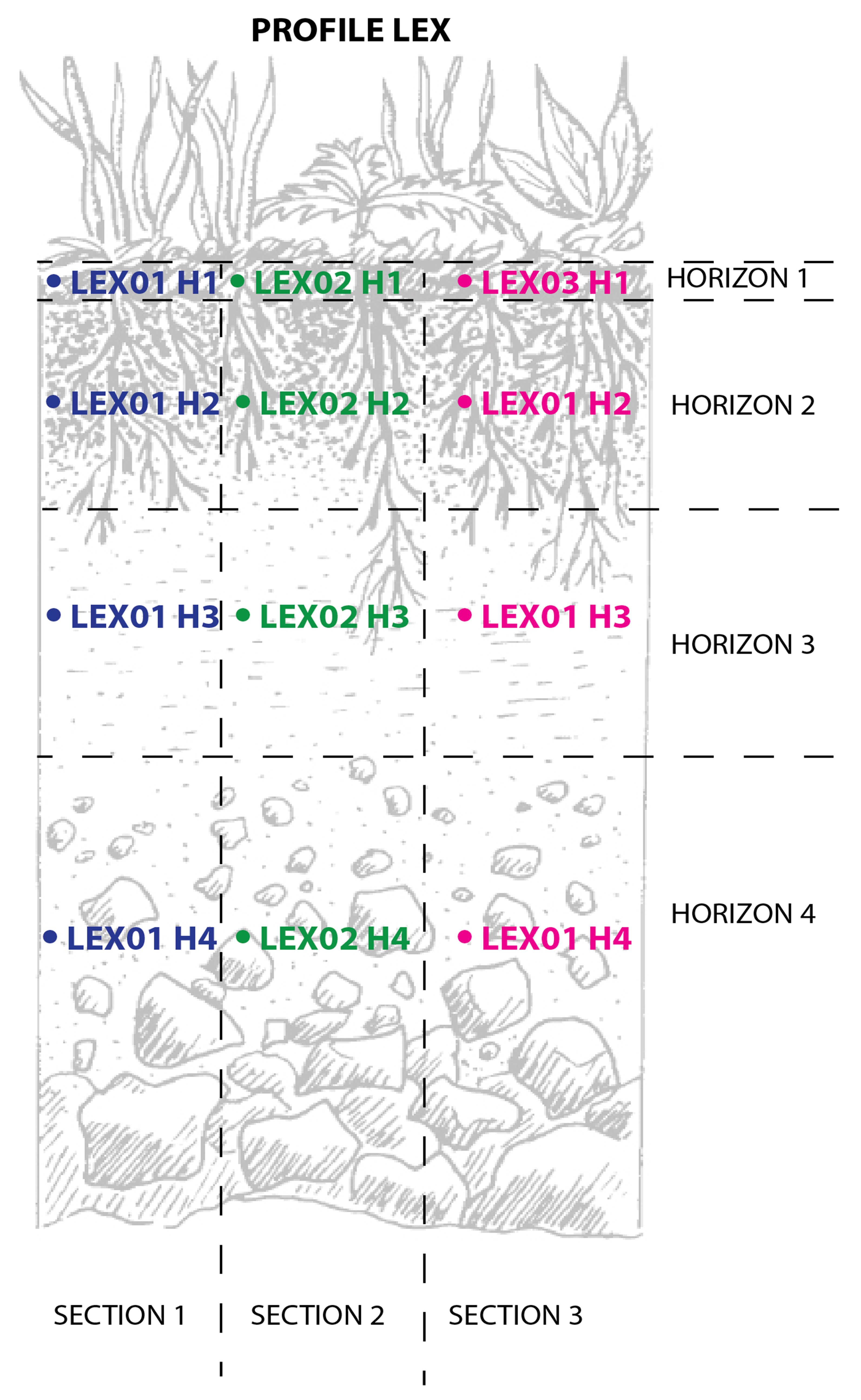

Supplement: Supplementary file 5 — Additional file 5: Figure S5. Sampling strategy. Soil sampling procedure and nomenclature used for biological samples for a ficticious profile LEX. [file 40659_2023_445_MOESM5_ESM.tif]
